# Texturing of Soy Yoghurt Alternatives: Pectin Microgel Particles Serve as Inactive Fillers and Weaken the Soy Protein Gel Structure

**DOI:** 10.3390/gels9060473

**Published:** 2023-06-08

**Authors:** Gabriela Itziar Saavedra Isusi, Johannes Marburger, Nils Lohner, Ulrike S. van der Schaaf

**Affiliations:** 1 Thermo Fisher Scientific, Pfannkuchstr. 10-12, D-76185 Karlsruhe, Germany; 2 Institute of Process Engineering in Life Sciences—Food Process Engineering, Karlsruhe Institute of Technology, Gotthard-Franz-Str. 3, D-76131 Karlsruhe, Germanynils.lohner7@gmail.com (N.L.)

**Keywords:** tribology, pectin, hydrocolloid, fermented gels, microgel, water-holding capacity, cross-linking, gel network, food texture, filler particles

## Abstract

Soy-based yoghurt alternatives were highly requested by consumers over the last few years. However, their texture does not always fulfil consumers’ demands as such yoghurt alternatives are often perceived as too firm or too soft, sandy, or fibrous. In order to improve the texture, fibres, for example, in the form of microgel particles (MGP), can be added to the soy matrix. MGP are expected to interact with soy proteins, creating different microstructures and, thus, different gel properties after fermentation. In this study, pectin-based MGP were added in different sizes and concentrations, and the soy gel properties after fermentation were characterised. It was found that the addition of 1 wt.% MGP influenced neither the flow behaviour nor the tribological/lubrication properties of the soy matrix, regardless of the MGP size. However, at higher MGP concentrations (3 and 5 wt.%), the viscosity and yield stress were reduced, the gel strength and cross-linking density decreased, and the water-holding capacity was reduced. At 5 wt.%, strong and visible phase separation occurred. Thus, it can be concluded that apple pectin-based MGP serve as inactive fillers in fermented soy protein matrices. They can, therefore, be used to weaken the gel matrix purposely to create novel microstructures.

## 1. Introduction

Fermented dairy-products, such as yoghurt, are part of the daily lives of many consumers. In recent years, consumers’ demand for vegan, vegetarian, and/or dairy-free food products increased for many reasons, such as dairy intolerances, environmental concerns, or animal well-being. This trend prompted the appearance of various plant-based yoghurt alternatives. Plant proteins from soy are one of the raw materials used most often to replace dairy proteins. However, the production of plant-based food alternatives is not just about replacing animal protein with plant protein. Instead, plant-based food alternatives are requested to have similar product properties in order to be accepted by consumers [1]. Therefore, the achievement of texture, viscosity, organoleptic, and sensory properties comparable to dairy-based yoghurts is the main technological challenge in the preparation of soy yoghurt.

Plant proteins, such as soy proteins, gel in the same manner as dairy proteins, i.e., due to the lack of electrostatic repulsion around their isoelectric point (pH 4.5–4.8). However, the resulting plant protein gels are often found to be “too firm” or “brittle” [2]. Their hard consistency or texture, which lacks creaminess, is not what consumers demand of yoghurt alternatives [3,4,5]. One reason for this difference in texture compared to dairy products is the strong cross-linking of the plant protein gel, resulting in small pores in which little water can be stored (low water-binding capacity) [6,7]. For this reason, strategies to mimic the dairy gel microstructure using plant-based ingredients are investigated for plant-based yoghurt alternatives [8].

Strategies to improve protein gel properties involve, for example, the addition of dietary fibre. It was noticed that very few authors addressed interactions between plant protein matrices and dietary fibres [9,10]. Most of the findings described were obtained in dairy yoghurts. Dietary fibre serves as a thickening agent and can increase the water-binding capacity of protein gels [4]. However, it was shown for dairy yoghurts that the addition of insoluble dietary fibre produces a sandy mouthfeel because insoluble fibre particles are not deformable or they are too large to have the desired effect [11,12,13]. In addition, insoluble dietary fibres may sterically hinder the formation of a protein network [14]. In contrast, soluble dietary fibres can enhance protein network formation. Low-esterified pectin is reported to interact with casein micelles and serve as a support for the formation of the protein network [15]. However, if the concentration of pectin is too high, the formation of the protein network is inhibited due to the electrostatic adsorption of pectin onto the casein micelles [16], which negatively affects the flow properties of the product [17]. In dairy yoghurts, mixtures of dietary fibres, such as inulin and pectin, were shown to have positive effects on product creaminess and consumer acceptance [15,18]. Thus far, only mixtures of several types of dietary fibres (e.g., pectin/inulin, orange fibre) were investigated regarding their effect on reduced fat dairy products. Moreover, complex systems of both soluble and insoluble fibres, such as orange fibre, can vary strongly in their fibre composition depending on batch and season. This issue makes intensive investigations into the exact composition necessary in order to thoroughly understand the observed effects on the gel properties [12]. For example, if more insoluble components are present in the used batch, the beneficial effect of soluble components will be masked, which may complicate the targeted texture modulation of plant-based yoghurt alternatives.

In summary, the influence of particulate, deformable, soluble dietary fibres in a plant protein matrix remains unclear. However, this is relevant information because there are several other approaches used to modulate the structure of fermented protein gels that are based on the addition of particles or colloids, such as protein aggregates, fat globules, or protein–polysaccharide complexes [19,20,21,22]. The decisive factor here is how the added colloids interact with the protein matrix and the make-up of their particle properties and distribution [23,24,25]. In our own preliminary work, it was shown [25] that fat particles affect the water-binding capacity the strength of the protein gel depending on their interaction with a β-lactoglobulin matrix. Particles that were not bound to the protein gel caused loss of strength and increased the occurrence of syneresis. In contrast, gels with bound particles showed significantly higher mechanical stability.

In addition to the mechanical characteristics, added particles can affect the tribological behaviour of the samples and, thus, influence product properties, especially the creaminess [12,20]. Laguna et al. [26] showed that tribological data differences are more suitable than rheological values for modelling sensory differences in full-fat and fat-free yoghurt, as an example of disperse systems. Some authors were able to show that tribological differences are measurable, although this was not evident in the rheological measurements [27]. Other authors already showed that the addition of particles can increase the friction values of a system [12]. Krzeminski et al. [20] showed that pectin–whey protein complexes can increase the creaminess of low-fat dairy products. This fact is due to the particulate nature of the complexes and the soft structure of the particles. By studying whey protein–pectin complexes in reduced-fat yoghurt, the researchers showed that small, soft particles produced lower friction in the mixed friction regime than large, hard particles. Here, the friction coefficient depends on the lubricating properties of the continuous phase and the elasticity of the particles. Large particles can hold less water compared to smaller ones because they are less integrated into the protein network. This outcome leads to increased friction values [12]. However, the use of particulate whey proteins is not feasible in vegan yoghurt alternatives.

In contrast, soft particles of cross-linked soluble dietary fibre, i.e., plant-based microgel particles (MGP) with defined deformability, could be a suitable option for this purpose. For this reason, the present work focuses on the use of microgel particles made from the soluble dietary fibre pectin as a way to modulate the rheological properties and the wate-binding capacity of fermented soy protein matrices. As soft, deformable structures, MGP are intermediate actors between polymers and particles in terms of their properties. Through a specific selection of the polymer type, its concentration, and the manufacturing process, MGPn can be produced in different shapes and sizes [28,29,30,31,32,33].

Generally, we can distinguish between top-down and bottom-up preparation methods [29]. In the top-down method, a polymer solution is made to gel under shear. In this process, it was possible to produce MGP in sizes ranging from 0.1 µm to 100 µm using conventional dispersing machines, such as high-pressure homogenizers or rotor stator systems. Our own research on the preparation of pectin-based MGPn (in top-down processes) showed that microgel particles, ranging from 17 μm to 137 μm in size, were comminuted to a size that depended on the energy input of the dispersing process [34].

To the best of our knowledge, the influence of dietary fibre-based MGP on the structure formation in and, thus, on the texture of plant protein gels, i.e., vegan yoghurt alternatives, was only described in one study [27]. In our previous work, we used MGP as fat stabilizers in soy protein yoghurts. We could show how the presence of both fat- and pectin-based MGP, soy protein, or a mixture of both were used as emulsifying agents in order to embed coconut oil droplets into the soy protein matrix. Even though the soy protein gels had the same formulation, different microstructures were obtained. These microstructures were found to affect the rheological and tribological behaviour of the soy gels. However, in this work, MGP were used as emulsifiers and interacted with the fat droplets.

Many questions regarding this complex particle–protein gel system remain open. We are most interested in the question of whether effects concerning the modulation of protein gel properties by particle addition known from Wiedenmann et al. [25] can be translated to plant protein matrices. Based on the literature, it is clear that pectin in its polymer form positively interacts with plant proteins such as soy [35]. We, therefore, hypothesize that pectin-based MGP will interact with the soy protein matrix, resulting in bound particles. As bound particles increase the mechanical strength and water-holding capacity of β-lactoglobulin gels [25], we expect comparable effects upon the addition of pectin-based MGPn in soy protein gels. Since the addition of dietary fibre improves the water-holding capacity of dairy gels, we assume an additional positive effect, even if pectin is added as MGP and not as “loose polymer” into the soy protein matrix. In order to test these hypotheses, pectin-based MGPn were prepared in a top-down process to yield differently sized MGPn. These MGPn were added to the soy matrix in different concentrations. The matrix was then fermented, and the rheology and water-holding capacity were investigated. This work aims to enable a better understanding of the texture modulation in plant protein yoghurt alternatives by shedding light on the interaction between soluble fibre and the plant protein matrix. Understanding these interactions can help us in formulating alternative yoghurt products with desirable textures.

## 2. Results and Discussion

### 2.1. Microgel Particle Properties

**Figure 1:**
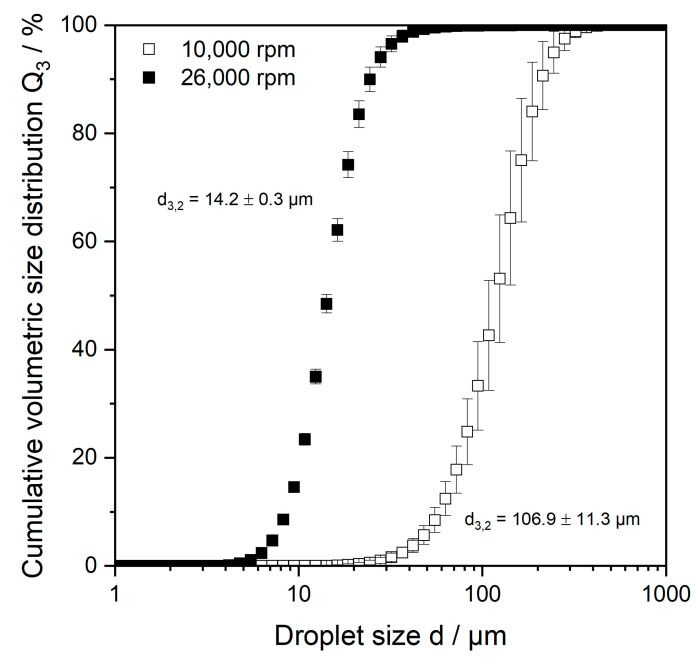
Cumulative volumetric droplet size distributions Q_3_ of 5 wt.% microgel particles in suspension. Microgels were comminuted using a colloid mill at two different rotating speeds. For comparison, Sauter mean diameter of depicted suspensions is stated.

The determination of the microgel particle size for samples with a target size of 1 µm was performed via dynamic light scattering. The particles had a z-average of 77.0 ± 12.8 nm and a polydispersity index of 2.64 ± 0.59. The size of the particles in the two larger microgel samples (100 and 10 µm) was determined through laser diffraction. The results are shown as volumetric cumulative distributions Q_3_ in Figure 1. The Sauter diameter of the distributions was taken as a parameter to verify that the target size of 10 and 100 µm was achieved. In Figure 1, it can be seen that the chosen process parameters led to MGP suspensions with narrow and monomodal size distributions. They have equivalent Sauter diameters of 14.2 ± 0.3 µm and 106.9 ± 11.3 µm, respectively, which are close to the target size.

The pH decrease during the fermentation of soy protein can affect the MGP charge. As possible interactions between MGP and protein also depend on the electrostatic charge of both molecules, the zeta potentials of MGP suspensions (MGP in water) were measured. For the measurements, pH-values 5, 4, and 3 were chosen, as they are the values at which soy proteins (glycinin and conglycinin) would coagulate to form the soy gel that composes the soy yoghurt [36]. The results are shown in Table 1.

The functional groups responsible for the surface charge of MGP are carboxyl groups (due to the low degree of methyl esterification of pectin). Although these groups are also involved in the gelation of pectin, it is unlikely that all carboxyl groups formed connecting zones with calcium ions. Free carboxyl groups could still remain in the MGP, giving them a strong negative surface charge, as measured by their ZP. The changes in the charge of MGP under acidic conditions can be explained by the charge state of the functional groups of MGP: decreasing the pH of the solution protonates the carboxyl groups, decreases the electrostatic surface charge of the MGP, and, thus, increases its zeta potential. Nevertheless, the surface charge remains negative over the studied pH range of interest.

### 2.2. Influence of the Addition of MGP with Different Particle Sizes on the Rheological and Tribological Properties of an SPI Matrix

**Figure 2:**
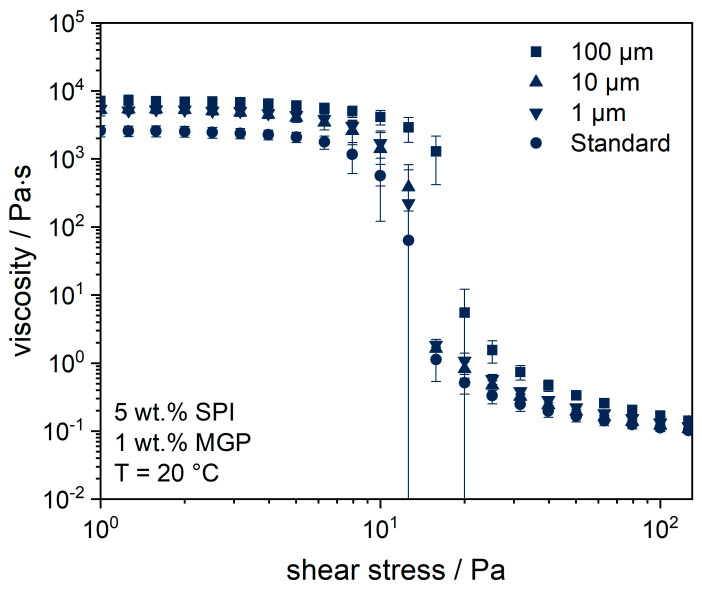
Viscosity curves of fermented soy protein gels, measured under increasing shear stress, with or without (Standard) addition of microgel particles of different sizes.

Possible MGP–protein interactions depend on the surface properties of the MGP, MGP size, and MGP concentration. In the first step, the influence of MGP size was investigated by adding MGP with a diameter of 1, 10, or 100 µm to the SPI matrix before fermentation. Rheological parameters were chosen as comparison parameters. The viscosity of stirred SPI gels with and without the addition of 1 wt.% MGP was measured after one day of storage at 5 °C (see Figure 2). A constant zero viscosity of the standard (without MGP) of about 2000 Pa∙s was seen in the range of up to τ ≈ 8 Pa. Thereafter, there was a rapid decrease in viscosity until it settled in the range of η ≈ 0.1 Pa∙s. The particle addition had a positive effect on the viscosity of the overall system. The largest effect was observed when the MGP size was 100 µm. Nevertheless, overall, there was very little difference between the samples. The 100 µm MGP sample showed an increased zero viscosity above 5000 Pa∙s. Thus, the particles increased the yield point of the SPI matrix. However, this result could be due to a steric effect. Larger MGP might have increased the yield point of the matrix because they disturbed the flow in the gap of the measurement geometry, which is evaluated as an increased resistance. Thus, no clear conclusion can be drawn on the interactions between pectin-based MGP and the SPI matrix.

The limited effect of MGP addition on the viscosity of the samples could be due to the MGP concentration. Krzeminski et al. [20] reported that pectin in dairy yoghurt can also weaken the aggregation of whey protein through steric hindrance, resulting in a slightly lower viscosity of the overall system compared to one without pectin addition. For this reason, further tests were made with higher MGP concentrations (see Section 2.3).

**Figure 3:**
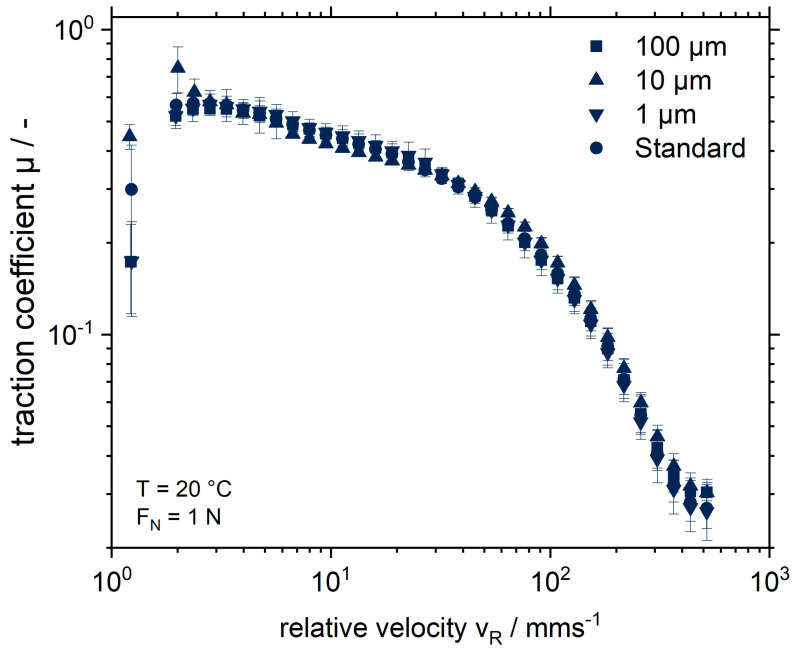
Stribeck curves of fermented soy protein gels with or without (Standard) addition of 1 wt.% microgel particles of different sizes.

Since the viscosity measurements shown above did not display strong effects, tribological measurements were conducted for samples containing 1 wt.% MGP (1, 10 or 100 µm). The aim was to verify whether findings valid for pectin–protein complexes in low-fat yoghurt, i.e., an increased friction for large particles [20], were also valid for pectin-based MGP in SPI gels. Figure 3 shows that neither the particles themselves nor their size have a measurable effect on the friction of the system under the given conditions. In the boundary friction range at v_R_ between 2 and 30 mms^−1^, the friction coefficient is in the range of 0.3 to 0.6. Similar values with μ ≈ 0.6 were found by Nguyen et al. [37] for fat-reduced and stirred soy yoghurt, as well as by Krzeminski et al. [20] for fat-reduced dairy yoghurt. Subsequently, due to the lubricating film formation, the transition to the mixed friction region occurs. Thus, the hypothesis that the MGP have a positive effect on the friction of the system could not be confirmed. A possible explanation for this finding is, again, the MGP concentration. Kieserling et al. [12] found no change in the friction coefficient when 0.1% orange fibres in the size range of 20 μm and 80 μm were added to yoghurt. However, for concentrations of 1 wt.%, the friction coefficient increased. Thus, the concentration of particles and pectin is a key variable influencing the friction values. In the experiments reported here, 1 wt.% MGP was used, which is a concentration that should result in measurable differences. However, the pectin concentration within the MGP is 2 wt.%, meaning that the proportion of pure pectin in the soy yoghurt is 0.02 wt.%. This figure is significantly lower than the value of 0.1 wt.%, which showed no significant influence on the friction values of yoghurt for orange fibres.

### 2.3. Influence of MGP Concentration on the Flow Properties and Water-Holding Capacity of the SPI Matrix

**Figure 4:**
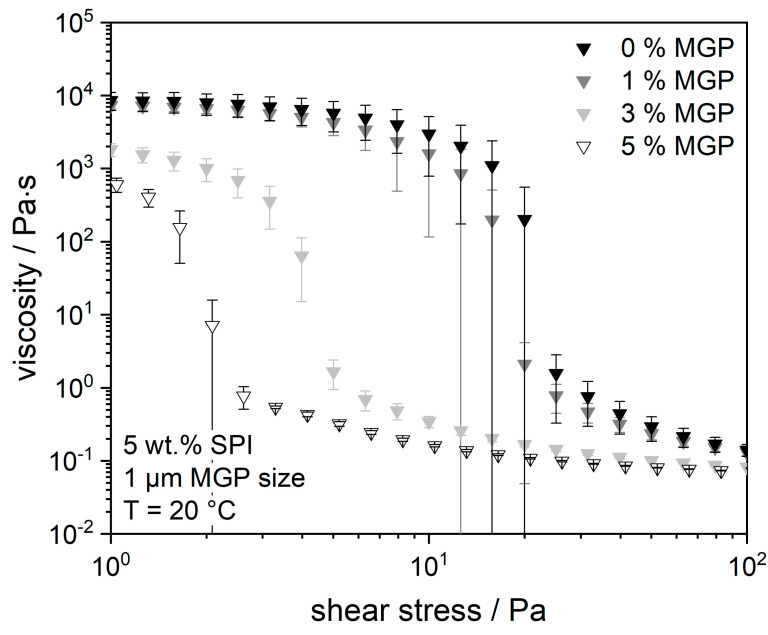
Viscosity curves of fermented soy protein gels, measured under increasing shear stress, with or without (Standard) the addition of different concentrations of microgel particles (1 µm of size).

Soy protein yoghurts with different concentrations of MGP were studied. The chosen MGP concentrations were 1 wt.%, 3 wt.%, and 5 wt.%, and the SPI concentration remained constant at 5 wt.% to ensure comparability with the previous results. Figure 4 shows the viscosity curves of the above samples.

It can be seen from the figure that the measuring points of both the yoghurts with 0 and 1 wt.% MGP are almost identical. From this result, it can be concluded that the addition of this MGP concentration does not lead to any change in viscosity, i.e., no change in the flow resistance of the overall system. On the one hand, the addition of the MGP does not strengthen the gel structure; on the other hand, it does not weaken it.

**Figure 5:**
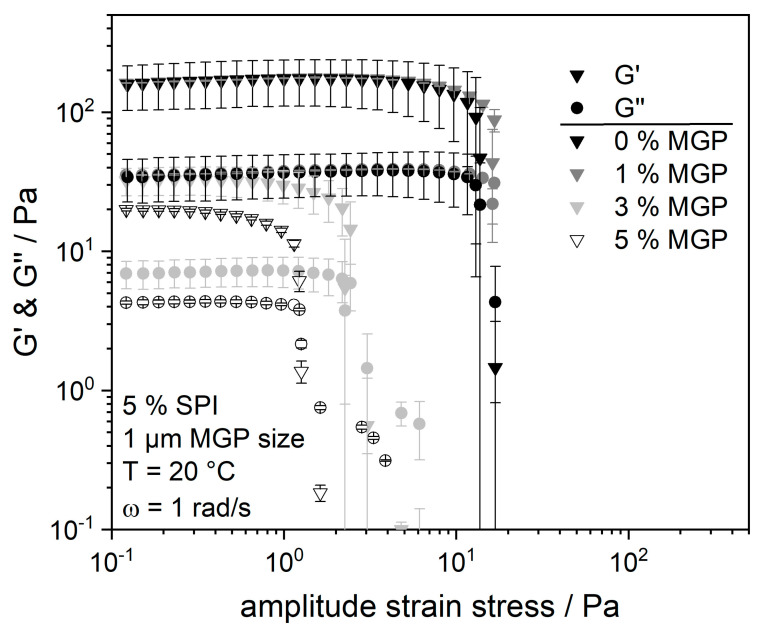
Amplitude test of fermented soy protein gels, measured at 20 °C and at an angular frequency of 1 rads^−1^. Different concentrations of microgel particles (1 µm of size) were added.

However, for higher MGP concentrations (3 and 5 wt.%), the addition resulted in a lower flow resistance of the samples. It could be seen by the eye that higher MGP concentrations hindered the formation of a complete three-dimensional protein network, most likely by preventing the proteins from fully agglomerating. This effect occurs more strongly with increasing MGP concentration, as can also be seen from the viscosity curve of the 5 wt.% MGP sample. A possible explanation for this outcome is the difference in the surface charge of MGP and SPI. MGP from amidated pectin are negatively charged at pH 5 and pH 4 (about −39 mV at pH 5). The IEP of SPI lies in this pH range; thus, the electrostatic charge of the molecules would approach zero. Thus, electrostatic interactions between protein and MGP occur only in a very limited form. Likewise, MGP repel each other—an effect that might interfere with protein aggregation. The more charged particles there are in the SPI matrix, the more obstacles there are to the formation of the protein matrix. To verify this fact, the degree of crosslinking within the protein matrix was determined using oscillatory measurements. Amplitude tests were performed to determine the moduli and the range of the linear viscoelastic region (LVE). The results of these tests are plotted in Figure 5.

Analogous to the results presented in Figure 4, it can be seen from Figure 5 that the addition of 1 wt.% MGP has no measurable effect on the viscoelastic properties of the SPI matrix. It can be concluded that the addition of amidated pectin-based microgel particles at this protein and MGP concentration does not lead to any change in protein crosslinking or in the stability of the protein gel structure. However, a significant change in the elasticity (G′ value) and, thus, in the degree of cross-linking of the protein matrix is observable at 3 and 5 wt.% MGP. In both cases, the LVE region was shorter, the cross-over point shifted, and both moduli (G′ and G″) were lower. These changes increase with increasing MGP concentration. Thus, the above hypothesis is confirmed: pectin-based MGP act as inactive fillers and prevent soy–protein coagulation.

**Figure 6:**
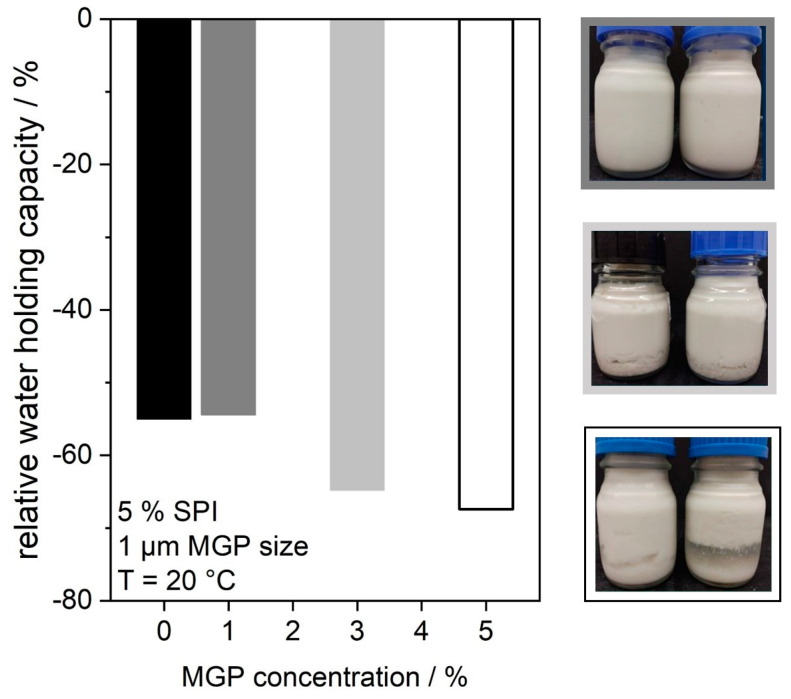
Relative water-holding capacity of fermented soy protein gels after centrifugation at 20 °C. Different concentrations of microgel particles (1 µm of size) were added. On right side, photographs of corresponding bulk samples are shown.

From our own previous studies, it is known that inactive filler particles do not only influence the gel strength of a protein matrix; rather, they can also negatively impact the water-holding capacity. This fact is presented in Figure 6 for the addition of different amounts of MGP to the fermented soy protein matrix. The results are shown in form of diagram depicting the relative water-holding capacity depending on the MGP concentration, as well as in the form of photographs of the corresponding samples. The diagram demonstrates how much water, relative to the total weighed-in mass, was lost after centrifugation. As expected, it can be seen that higher concentrations of MGP have a negative impact on the water-holding capacity of the samples. While the addition of 1 wt.% MGP does not reduce the WHC compared to the standard sample, the WHC decreases at 3 wt.% MGP and is lowest at 5 wt.%. Visual inspection confirms these results. At 1 wt.% MGP, the bulk samples appear homogeneous. However, at 5 wt.% MGP, the formation of a homogeneous protein network was hindered to such an extent that water was expressed from the protein matrix at rest, i.e., without any mechanical stress, and phase separation was observable.

## 3. Conclusions

The aim of this work was to investigate the texture-changing properties of pectin-based MGP in soy-based yoghurt alternatives. In particular, the influence of the particle size and concentration on the rheological and tribological properties and on the water-holding capacity was to be investigated because these properties correlate with consumer-relevant product properties, such as creaminess, homogeneity etc. In order to investigate the texture-changing properties of MGP, a model system based on soy protein isolate was developed. Size-defined microgel particles of approximately 1, 10, and 100 µm made of apple pectin were added to model yoghurt alternative in different concentrations before fermentation. After fermentation, the samples were characterised for the named properties.

It could be shown that at a concentration of 1 wt%., MGP did not have any influence on the rheological and tribological properties of soy-based yoghurt alternatives, regardless of their size. This outcome was unexpected because smaller particles at the same concentration have an overall larger active surface area. This finding makes it more likely that interactions, be they positive or negative, with protein molecules take place. Instead, MGP weight concentration was found to negatively impact the soy gel properties. At 3 wt.%, and even more so at 5 wt.%, MGP were found to weaken the soy protein gel. The added particles weakened the internal cohesion and, thus, the gel strength of the entire system by creating less strong protein–protein connections. This finding was demonstrated in terms of a reduced cross-linking density. Product properties were also affected in that flow behaviour and water-holding capacity were reduced. At the highest MGP concentration, visual phase separation occurred. Thus, it could be concluded that MGP acted as inactive fillers in fermented soy protein matrices. The addition of pectin-based MGP to fermented soy protein matrices can be used to purposely weaken the matrix. These findings can, thus, be used to modulate soy gel properties in a targeted manner and aid the development of novel vegetarian/vegan food products.

## 4. Materials and Methods

### 4.1. Materials

Low methyl-esterified amidated apple pomace pectin was gifted by Herbstreith and Fox (Neuenbürg, Germany). The pectin had a degree of esterification of 24%, a degree of amidation of 24%, and a galacturonic acid content of 91%, according to the supplier’s specifications. Calcium chloride di-hydrate (analytical grade) was obtained from Merck KGaA (Darmstadt, Germany). Soy protein isolate (SPI) was kindly provided by Danisco Deutschland GmbH. Starter cultures (*Lactobacillus bulgaricus* and *Streptocuccus thermophilus*) were purchased from Metafood GmbH (Frankfurt, Germany). D-Saccharose was purchased from Carl Roth (Karlsruhe, Germany).

### 4.2. Preparations of Pectin Solution

The amidated apple pomace pectin solution, with a pectin mass concentration of 2 wt.%, was prepared by dissolving 4 g pectin in 196 g of demineralised water in a 400 mL beaker. Pectin was dissolved using a magnetic stirrer (IKA^®^ Werke GmbH & Co. KG, Staufen, Germany) at a temperature of 70 °C. After fully dissolving the pectin, the solution was left to cool down to room temperature.

### 4.3. Preparation of Pectin MGP Suspensions

The pectin solution prepared as described above was used for the preparation of MGP suspensions with a 50 wt.% MGP concentration, according to the method described by Saavedra Isusi et al. [36]. Gelation was triggered by adding a 40 mM CaCl_2_ solution to the pectin solution under constant shearing with a high-shear mixer Ultraturrax T-25 digital (IKA^®^ Werke GmbH & Co. KG, Staufen, Germany) at a rotational speed of 13,000 rpm for 3 min.

Subsequently, the obtained stock microgel suspension was diluted to 5 wt.% microgel concentration with demineralised water and further comminuted to give MGP suspensions of different particle sizes. Different particle sizes were achieved via mechanical energy input, as described previously [38]. For the production of microgel particles of 10 and 100 µm, a colloid mill (IKA magic Lab, module MK, IKA-Werke, Staufen Germany) was used. For the production of the 1 µm microgel particles, a high-pressure homogenizer (Microfuidizer M-110 EH, Microfluidics, Newton, MA, USA) was used. The precise process parameters used are listed in Table 2.

### 4.4. Determination of MGP Size and Zeta-Potential

To corroborate that the produced MGP had the targeted size, either their particle size or hydrodynamic diameter was measured. The size of the microgel particles produced (100 and 10 µm) was determined via laser diffraction. The measurement method is based on the Mie theory for light scattering, which was developed for spherical particles. However, microgel particles are not perfect spheres; thus, the measured values were considered as having equivalent diameters and are shown as volumetric cumulative distribution Q_3_. The Sauter diameter of the distributions was taken as a parameter to verify that the target size was achieved. The particle size distribution (PSD) of the prepared microgel suspension was determined via static laser light scattering using a HORIBA LA-950 Particle analyser (Retsch Technology, Haan, Germany). The refractive indices were set at *n* = 1.5470 + 0.01 i for pectin and *n* = 1.333 for water for all samples. All measurements were conducted in triplicate at room temperature.

The determination of the microgel particle size for samples with 1 µm was performed via dynamic light scattering. The hydrodynamic diameter (z-average) of MGPs in suspension was determined via dynamic light scattering with a particle size analyser Horiba Nanopartica SZ-100 (Horiba Scientific, Kyoto, Japan). Samples were measured at least 10 times. Measurements were conducted at a scattering angle of 173° and at 22.0 ± 1 °C. The particles had a z-average of 77.0 ± 12.8 nm.

The zeta potential of MGP suspensions containing 1 wt.% MGPs was determined using the particle size analyser Horiba Nanopartica SZ-100 (Horiba Scientific, Kyoto, Japan), at pH-values 5, 4, and 3, without adjusting the electric conductivity (EC) of the water phase. Three measurements of 10 runs were conducted at 25 °C for each solution.

### 4.5. Preparation of Soy Protein Gels

Soy protein gels were prepared through fermentation. Six formulations containing different amounts of MGP, as well as one control sample without MGP, were investigated (see Table 3). Samples containing MGP were prepared by mixing concentrated soy protein solutions with MGP suspensions. The concentration of the soy protein solutions was chosen to ensure that the protein concentration in the final samples (after mixing with MGP suspensions) was always the same.

The soy protein solutions were all prepared by dissolving SPI powder in demineralised water at 60 °C for at least 30 min. After that, the SPI solutions were pasteurised via heating to 80 °C and holding the temperature for 1 min. Next, the protein solutions were allowed to cool down to 43.5 °C. Once that temperature was reached, the required amount of MGP suspension for the respective sample was added to the SPI solutions. Afterwards, 1 wt.% sucrose and 0.5 wt.% of the starter cultures were added. The different samples were poured into individual commercially available yoghurt cups, which were sealed with aluminium foil. The samples were let to ferment in a water bath at 43.5 °C for approximately 16 h until the pH of the samples reached 4.3 ± 0.2. Thereafter, the pot-set SPI gels were stirred at 3000 rpm for 30 s with a high-shear mixer and cooled at 5 °C for at least 24 h prior to analysis. All formulations were prepared at in triplicate.

### 4.6. Tribological Measurements

All tribological measurements were performed using a HAAKE Mars rheometer (Thermo Electron GmbH, Karlsruhe, Germany) in triplicate. A 125 mm diameter steel ball and three polydimethylsiloxane (PDMS)-based silicone pins (Sylgard 184, Dow Inc., Midland, MI, USA) were chosen as tribo-pairs. The pins were cut out from a cast plate. The coefficient of friction between tribo-pairs and sample was plotted versus the rotational velocity from 0.1 mms^−1^ to 600 mms^−1^, at a normal force of 1 N and a temperature of 20 °C, where 100 measurement points were taken. Each individual preparation of a sample was analysed at least 5 times.

### 4.7. Rheological Measurements

All rheological analysis were conducted using a stress-controlled Physica MCR 301 rotational Rheometer (Anton Paar, Graz, Austria). These analyses were performed using a plate-plate measurement system with a plate diameter of 25 mm (PP25). The gap width was 1 mm.

Amplitude sweeps were performed at an angular frequency of ω = 1 rad s^−1^ over a range of amplitude stresses from 1 to 100 Pa. The amplitude stress was increased logarithmically, and 11 measurement points were taken for each decade. All amplitude sweeps were conducted in duplicate for each sample.

Viscosity measurements were realised at a shear stress from 0.1 to 100 Pa. The shear stress was increased in a logarithmic manner, while 10 measurement points were taken for each decade, with a measurement point duration of 10 s. The viscosity measurements were performed in triplicate for each sample.

### 4.8. Water-Binding Capacity Measurements

The water-binding capacity of the different soy protein gels was measured by placing approximately 30 g of the different samples into a centrifuge tube and determining the exact mass. The samples were centrifuged for 10 min at a rotational speed of 5000 rpm. The excess water after the centrifugation was weighed, and the water-binding capacity calculated with the mass of the sample before centrifugation and the mass of the excess water. The water-binding capacity was determined in triplicate for each sample.

### 4.9. Statistical Analysis

Each sample preparation was carried out in triplicate. If not specified otherwise, all analyses were conducted at least three times per independent test. All data was assessed via a multifactorial analysis of variance (ANOVA) and a Tukey test as post hoc test. Dissimilarities in samples were considered statistically relevant at a level of *p* ≤ 0.05. The software OriginPro 2019 (OriginLab Corp., Northampton, MA, USA) was used for the statistical analysis, calculation of averages, and standard deviations.

## Figures and Tables

**Table 1:** Zeta potential of microgel particles in suspension, depending on pH.

pH	Zeta Potential/mV
3.0	−17
4.0	−33.7
5.0	−39.1

**Table 2:** Process parameters used for production of MGP from amidated pectin.

MGP Size	Dispersing Device	Rotational Speed/Pressure	Time/Passes
100 µm	Colloid mill	10,000 rpm	1 min
10 µm	Colloid mill	26,000 rpm	5 min
1 µm	High-pressure homogenizer	400 bar	2

**Table 3:** Composition of investigated samples.

Sample Name	SPI Powder	MGP Size	MGP Suspension Content	Water Content
Reference; 5% SPI	25 g	-	-	475 g
1% MGP; 5% SPI	25 g	1 µm	100 g	375.5 g
1% MGP; 5% SPI	25 g	10 µm	100 g	375.5 g
1% MGP; 5% SPI	25 g	100 µm	100 g	375.5 g
3% MGP; 5% SPI	25 g	1 µm	300 g	175.5 g
5% MGP; 5% SPI	25 g	1 µm	500 g	-

## Data Availability

The data presented in this study are available on request from the corresponding author.
